# Dynamic Changes of Soluble Fas and IL-2/IL-10 in serum and Fas Expression in Lung in the Rats of Acute Necrotizing Pancreatitis

**DOI:** 10.4021/gr2008.11.1238

**Published:** 2008-11-20

**Authors:** Jian Xin Zhang, Jiang Tao Yin, Lei Cui, Sheng Chun Dang

**Affiliations:** a Department of General Surgery, Affiliated Hospital of Jiangsu University, Zhenjiang 212001, Jiangsu Province, China

**Keywords:** acute necrotizing pancreatitis, Fas, lung injury

## Abstract

**Background:**

To investigate the dynamic changes of serum IL-2, IL-10, sFas and IL-2/IL-10 in a rat model with acute necrotizing pancreatitis (ANP). To explore the role of Th1/Th2 polarization and the Fas expression in the lung of rats with ANP.

**Methods:**

A total of 64 Sprague-Dawley rats were randomly divided into normal control group and ANP model group. ANP models were induced by injection of 50 g/L sodium taurocholate (4 mL/kg) under the pancreatic membrane. In the normal control group, the rats received isovolumetric injection of 9 g/L normal saline solution. The blood samples in each group were obtained via superior mesenteric vein for measuring IL-2, IL-10 and soluble Fas. The levels of IL-2, IL-10 and soluble Fas were determined by ELISA. The severity of lung injury was evaluated by pathologic score. The expression of Fas in lung was measured by immunohistochemistry.

**Results:**

In the ANP model group, levels of serum IL-2 were significantly higher than those of control group (*P* < 0.01), and peaked at 6 hours; levels of serum IL-10 were significantly higher than those of control group at 6 and 12 hours (*P* < 0.01); the ratios of IL-2/IL-10 were significantly higher than those of control group at 0.5 hours and 2 hours, however, they were significantly lower than those of control group at 6 hours, (*P* < 0.01), and returned to the normal level (*P* > 0.05). In Fas/APO-1 assay, there was no significant difference between the two groups. The pathological changes were aggravated significantly in model group compared with the control group. Immunohistochemistry stain showed Fas expression was absent in normal pulmonary tissue, whereas in pulmonary tissue Fas expression gradually increased 0.5 hours after induction of pancreatitis, and reached their peaks at 12 hours.

**Conclusions:**

Fas are involved in the pathogenesis of pancreatitis associated lung injury, the mechanism might be related to the Fas mediated T helper cell apoptosis.

## Introduction

Acute necrotizing pancreatitis (ANP) is characterized by inflammatory and necrotic events, which follows the initial intra-acinar injury involving enzyme activation and disruption of the acinar cytoskeleton. ANP is often complicated by lung injury. However, its pathogenesis remains unclear. It is known that ANP involves a complex array of mediators that can initiate and amplify the systemic inflammatory response (SIRS).This can lead to the failure of distant organ systems, such as the lungs, heart and kidneys(1-3), among which, pulmonary failure is most common(4-7).

Recent studies indicate that during the pathogenesis of ANP, apoptosis plays an important role in the worsening of pancreatitis(3).The Fas system was originally characterized as a key mechanism for inducing apoptosis in immune cells. In the present study, we performed immunohistochemistry of apoptosis-related protein Fas and investigated dynamic changes of serum IL-2, IL-10, and sFas in rats with ANP.

## Materials and Methods

### Animals

Adult Sprague-Dawley rats of both sexes weighing 250 - 300 g were provided by the Laboratory Animal Center of Jiangsu University. The animals were fed with standard rat chow and water *ad libitum*. The rats were allowed to acclimatize to our laboratory conditions for 1 week and then subjected to mesh stainless-steel cages at a constant temperature (21 ± 1°C) in a 12 hour day/night cycle. The animals were fasted for food for 12 hour before the experiments but had free access to water. Animal care and experimental procedure were performed in accordance with the guidelines for Animal Experimentation of Jiangsu University with the approval of the Institutional Animal Care and Use Committee.

### Experimental design

The animals were randomly divided into control group (C group) and ANP group (P group) with 32 rats in each. Each group was further divided into 0.5, 2, 6 and 12 hour subgroups. The mortality in the present series was not calculated and the surviving rats were used. In the model group, the rats were anesthetized with an intraperitoneal injection of sodium pentobarbital (50 mg/kg body weight), then were infused with sodium taurocholate (4 mL/kg, Na-Tc, Sigma) in the pancreatic membrane to induce ANP model as previously described(8). After 30 min, pancreatic edema and dotted bleeding occurred. Normal control group received isovolumetric injection of 9 g/L physiological saline solution using the same method. Animals in each group were sacrificed at 0.5, 2, 6 and 12 hour after infusion for further examination. Left lung and pancreas were removed immediately and fixed in paraformaldehyde solution, and paraffin-embedded for pathologic analysis. The pathologist was blinded to sample groupings.

### Serum levels of IL-2, IL-10 and sFas

Serum levels of IL-2, IL-10 and sFas were measured by double antibody sandwich ELISA according to the manufacturer's protocol (Shanghai Senxiong Technology Enterprise Co., Ltd.). The optical density of each well was determined within 30 min using a microplate reader (492 nm).

### Pathological examination

The whole pancreas and left lung were obtained and promptly fixed in 40 g/L phosphate-buffered formaldehyde for further studies. Paraffin-embedded tissue sections (5 µm thick) were stained with hematoxylin and eosin. Histopathologic analysis of lung was performed according to the inflammatory scoring system(9): 0-III (absent, 0; mild, I; moderate, II; severe, III).

### Immunohistochemistry

After embedding in paraffin, sections in 5 µm thickness were immersed into xylene twice with 5 minutes each, followed by immersion in 100% ethanol immersion twice 3 minutes each, and then in 95% ethanol. Slides were rinsed for 30 seconds using deionized water and then immersed in deionized water twice for 5 minutes each. To detect Fas expression, heat-induced Ag retrieval was performed using 0.01mol/L citrate buffer (pH 6.2) and 10 minute slide immersion into 95 °C water bath. Immunoenzyme double staining of pulmonary tissue was performed using DAKO EnVision Doublestain System. The sections were then counterstained using hematoxylin before study.

### Statistical analysis

Results were expressed as mean ± SD except for the date of the grading of pulmonary mucosal injury. The results were analyzed using the post Hoc test. Differences of grading of lung injury were determined using the non-parametric Mann-Whitney test. Differences were considered to be statistically significant when *P* values were less than 0.05. Statistical analysis was performed using SPSS 11.5 for Windows.

## Results

### Serum IL-2 level

At 0.5 h after injection of 50 g/L sodium taurocholate, serum IL-2 levels in P group were higher than those in C group. From 0.5 hour, there was a significant difference between P group and C group (*P* < 0.01, Table 1).

**Table 1:** Serum IL-2 level (pg/ml) in each group

Group	0.5 hour	2 hour	6 hour	12 hour
C	2.79 ± 0.51	2.93 ± 0.89	4.81 ± 1.23	3.41 ± 0.72
P	3.53 ± 0.62^*^	4.35 ± 1.11^*^	6.94 ± 1.55^*^	4.80 ± 1.10^*^

^*^P < 0.01 vs. C group. C: control group; P: acute necrotic pancreatitis group.

### Serum IL-10 level

Upon stimulation, the release of IL-10 was significantly increased in the P group as compared with that of C group at 6 and 12 hours (*P* < 0.01, Table 2). There was no statistical difference in concentrations of IL-10 between P and C group at 0.5 and 2 hour.

**Table 2:** Serum IL-10 level (pg/ml) in each group

Group	0.5 hour	2 hour	6 hour	12 hour
C	54.61 ± 15.81	141.15 ± 40.21	89.18 ± 32.52	77.15 ± 22.60
P	47.34 ± 14.62	156.12 ± 43.10	494.98 ± 11.23^*^	93.28 ± 25.81^*^

^*^P < 0.01 vs. C group. C: control group; P: acute necrotic pancreatitis group.

### Serum IL-2/IL-10 ratio

As shown in Table 4, the values of IL-2/IL-10 were higher significantly in P group than those of C group at 0.5 and 2 hour, and it were significantly lower than those of C group at 6 hour (*P* < 0.01) and returned to the control level at 12 hour (*P* > 0.05) (Table 3).

**Table 3:** Serum IL-2/IL-10 ratio in each group

Group	0.5 hour	2 hour	6 hour	12 hour
C	0.05 ± 0.01	0.02 ± 0.01	0.05 ± 0.02	0.04 ± 0.01
P	0.07 ± 0.02^*^	0.03 ± 0.01^*^	0.01 ± 0.01^*^	0.05 ± 0.02

^*^P <0.01 vs. C group. C: control group; P: acute necrotic pancreatitis group.

**Table 4:** Fas/APO-1 level (pg/ml) in each group

Group	0.5 hour	2 hour	6 hour	12 hour
C	3.16 ± 0.75	4.05 ± 1.08	5.93 ± 1.52	4.62 ± 1.23
P	3.31 ± 0.80	4.32 ± 1.11	5.41 ± 1.47	4.44 ± 1.16

C: control group; P: acute necrotic pancreatitis group.

### Serum Fas/APO-1 level

In Fas/APO-1 assay as illustrated in Table 4, there was no significant difference between P group and C group.

### Pathologic examination of lung and pancreas

After induction of ANP model, pancreas showed mild edema and congestion. Two hours after induction of the model, typical pathologic changes were found in ANP, such as a large number of inflammatory cells, necrosis of adjacent fat tissues, interstitial edema, parenchyma hemorrhage and necrosis, large amount of ascites, with time, these changes became severer. The pulmonary pathological changes were aggravated significantly in the P group, the histopathologic scores were higher in the P groups than that of the C groups throughout the experimental course (*P* < 0.01) (Table5).

**Table 5:** Pulmonary tissues injury in each group

Group	0.5 hour				2 hour				6 hour				12 hour			
	0	I	II	III	0	I	II	III	0	I	II	III	0	I	II	III
C	8	0	0	0	7	1	0	0	7	1	0	0	7	1	0	0
P	2	5	1	0^*^	1	5	2	0^*^	0	4	3	1^*^	0	3	4	1^*^

^*^P<0.01 vs. C group. C: control group; P: acute necrotic pancreatitis group.

### Immunohistochemistry

**Figure 1:**
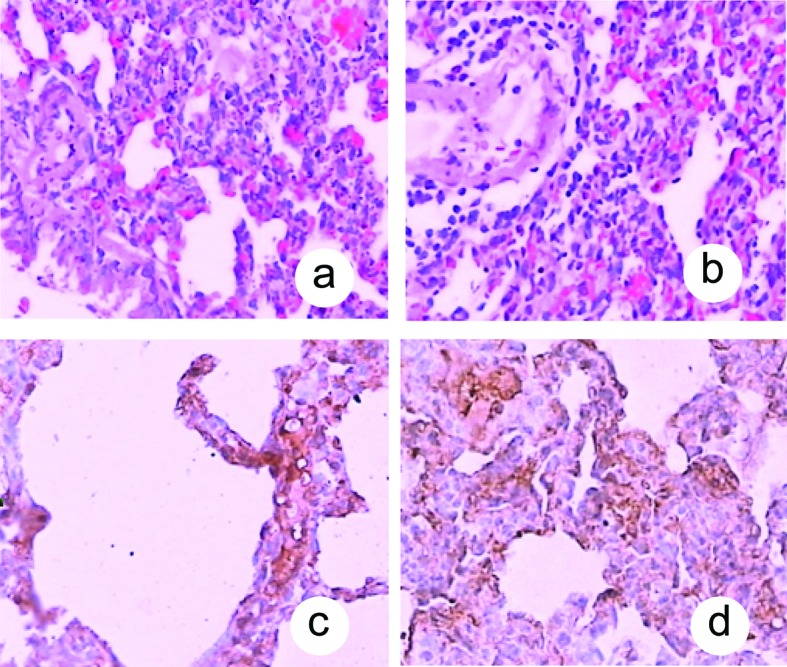
Histological changes in pulmonary tissue after induction of ANP. A: Lung tissue in the P group at 0.5 hour (HE, original magnification ×100); B: Lung tissue in the P group at 12 hour (HE, original magnification ×100); C: Fas had a lower expression in pulmonary tissue in the P group at 0.5 hour (original magnification ×400); D: Fas had a higher expression in pulmonary tissue in the P group at 12 hour (original magnification ×400).

Fas had a lower expression in pulmonary tissue in the P group at 0.5 hour and higher expression at 2, 6 and 12 hour. Fas expression in pulmonary tissue gradually increased after 0.5 hour then reached their peaks at 12 hour. In the C groups, Fas were not detected in any part of pulmonary tissue (Fig. 1).

## Discussion

Acute pancreatitis is a sudden inflammation of the pancreas that may be mild or life threatening that usually subsides. Although usually self-limiting, 10% to 20% of afflicted patients will progress to severe pancreatitis. The mortality rate among patients with severe pancreatitis may approach 30% when they progress to multiple organ failure(10). The ANP is often complicated by lung injury.

Apoptosis play an important role in the pancreatitis. The Fas system is a widely recognized apoptosis signal transduction pathway in which a ligand-receptor interaction triggers the cell death pathway(11). The Fas system has been implicated in the control of the immune response and inflammation, the response to infection, and death of parenchymal cells in several organs(12-14), which is involved in maintaining homeostasis in various systems, including maintenance of peripheral T cell(15). Recent research has demonstrated that the T-helper 1 (Th1) to T-helper 2 (Th2) immune deviation is beneficial to mucosal immunity(16, 17). A key component of the mucosal immune defense against pathogens is mediated by CD4+ T lymphocytes that can differentiate into functionally distinct subsets(18). While as the Th1 cells secrete the cytokines IL-2, IFN-γ, TNF-α and TNF-β, the Th2 cells secrete IL-4, 5, 6, 10, and 13. In the current study, we used IL-2 levels as a marker of Th1 response and IL-10 as a marker of Th2 response. In this study, the role of the Fas-mediated cell death pathway in lung injury was assessed and dynamic changes of serum IL-2, IL-10, IL-2/ IL-10 and sFas in rats with ANP were investigated.

According to immunohistochemistry results, Fas had a lower expression in pulmonary tissue in the ANP group at 0.5 h and had a higher expression after 2, 6 and 12 hours. In the control group, Fas were not detected in any section of pulmonary tissue.

Previous study has shown that IL-10 is a kind of important anti-inflammatory cytokine and plays a role of self-defense mechanism, limiting the intensity of inflammatory process(19-22). However, the effect of IL-10 level in the course of acute pancreatitis is still not clear(23-25). IL-10 is a powerful Th2 cell cytokine produced by lymphoid cells. A marked activation of immune system may be observed in patients with AP, which is balanced between pro- and anti-inflammatory cytokines in patients with mild AP but not in severe AP. A reduced functional reserve for the synthesis of IL-10 may be observed in patients with severe AP, which might lead to a worst prognosis(26-29).

From the results of this study, we found that the release of IL-10 was significantly increased in the ANP group as compared with that of control group at 6 and 12 hours. Serum IL-2 level in the ANP group were higher than those in control group. From 0.5 h, there was a significant difference between ANP group and control group. The IL-2/IL-10 ratio was significantly increased in the ANP group as compared with that of control group at 0.5 and 2 hours, suggesting that a pro-inflammatory response was predominant at these periods; IL-2/IL-10 ratio was significantly lower in ANP group at 6 hours, this suggested that an anti-inflammatory response was predominant at this time. In sFas assay, there was no significant difference between ANP group and control group. Interestingly, IL-2, IL-10 and sFas levels were moderate at 0.5 hours, peaked at 6 hours and decreased at 12 hours. In our model, the lung injury, which was assessed according to the standard of pathological examination, was closely paralleled with Fas expression and dynamic changes of IL-2/IL-10.

In conclusion, the abnormal apoptosis of Fas can significantly affect the cytokines. Fas are involved in the pathogenesis of lung injury in ANP. The mechanisms of Fas might be related to their mediation of T helper cell apoptosis.
